# Halofuginone is a Molecular Glue Degrader of Integrin *β*4

**DOI:** 10.1002/advs.202515970

**Published:** 2026-03-24

**Authors:** Wei Gong, Di Yang, Junrui Tian, Yufei Chai, Tiantian Yu, Qingnan Wu, Yan Wang, Weimin Zhang, Qimin Zhan

**Affiliations:** ^1^ Peking University‐Yunnan Baiyao International Medical Research Center Beijing China; ^2^ State Key Laboratory of Molecular Oncology Key laboratory of Carcinogenesis and Translational Research (Ministry of Education/Beijing) Laboratory of Molecular Oncology Peking University Cancer Hospital & Institute Beijing China; ^3^ Research Unit of Molecular Cancer Research Chinese Academy of Medical Sciences Beijing China; ^4^ Sinovac Biotech Co., Ltd. Beijing China; ^5^ School of Basic Medical Sciences Peking University Health Science Center Beijing China; ^6^ Shenzhen Bay Laboratory Shenzhen China

**Keywords:** Halofuginone, Integrin *β*4, Molecular Glue, CRL4B^WDR18^

## Abstract

Integrin *β*4 **(gene name: ITGB4)** overexpression is associated with aggressive phenotypes and poor prognosis across multiple solid tumors. Despite its clinical significance, therapeutic strategies targeting integrin *β*4 remain underdeveloped. Targeted protein degradation technology, particularly molecular glue degraders, offer a promising approach for eliminating oncoproteins. However, the serendipitous discovery of molecular glues and limited exploration of substrate receptor have largely hindered the rational development of molecular glue degraders. In this study, through high throughput screening from a natural product library, we identified halofuginone (HF) as a potential molecular glue that promotes integrin *β*4 degradation via the CRL4B^WDR18^ E3 ubiquitin ligase complex. HF administration markedly disrupted the aggressive progression of tumor cell both in vitro and in vivo. In summary, our study not only establishes HF as a promising degrader of integrin *β*4 but also demonstrates the utility of natural product screening for discovering molecular glue degraders, providing a novel therapeutic strategy for targeting oncogenic transmembrane proteins.

## Graphical abstract

Integrin β4 overexpression is linked to aggressive tumors and poor prognosis, but targeted therapies are lacking. We identified halofuginone (HF) as a molecular glue degrader that promotes integrin β4 degradation via the CRL4B^WDR18^ E3 ligase. HF suppresses tumor progression in vitro and in vivo, offering a new strategy for targeting oncogenic transmembrane proteins.

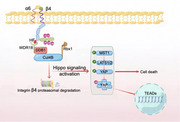

## 1 Introduction

Integrins are proteins crucial to a wide range of biological functions due to their pivotal roles in cell adhesion and signal transduction. Their ubiquitous distributions and transmembrane characters afford integrins critical roles in maintaining cell proliferation, migration and invasion of cancer cells [1], and in regulating cell differentiation, angiogenesis, epithelial‐to‐mesenchymal transition, and even therapeutic outcomes [2, 3]. These properties make integrins attractive prognostic indicators and therapeutic targets for various cancer types. Emerging strategies have successfully targeted integrins *α*IIb*β*3, *α*4*β*7, *α*4*β*1 and *α*L*β*2 for cardiovascular diseases, inflammatory bowel disease/multiple sclerosis and dry eye disease, respectively [4]. Among all integrins, integrin *β*4 heterodimerizes with integrin *α*6 to form a laminin receptor that mediates the hemidesmosomes formation [5]. In tumor cells, *α*6*β*4 promotes invasion by activating the phosphatidylinositol‐3 kinase pathway. This invasive process requires *α*6*β*4 re‐localization from a stable adhesive structure linked to intermediate filaments to dynamic motile structure linked to the actin cytoskeleton [6], potentially making it intractable to conventional pharmacological means. Structurally, integrin *β*4 is characterized by a uniquely large cytoplasmic tail, which includes two pairs of fibronectin type III domains separated by a connecting segment [6]. Accumulating evidence demonstrates high integrin *β*4 expression in many kinds of solid tumors, with high levels correlating with aggressive phenotypes and poor prognosis in lung cancer, breast cancer, pancreatic cancer, gastric cancer and cervical cancer [7, 8, 9]. This suggests integrin *β*4 confers an evolutionary function during tumor progression and represents a potential therapeutic target.

Targeted protein degradation (TPD) has emerged as a powerful strategy to eliminate cancer‐associated proteins, with proteolysis‐targeting chimeras (PROTACs) and molecular glues constituting the primary classes of TPD technologies [10]. Unlike PROTACs, molecular glue typically behaves as indivisible structures rather than hybrids of targeting proteins and E3 ligase ligand joined by a suitable chemical linker. Critically, molecular glue mediated protein degradation requires no pre‐existing ligand‐binding pocket on the targeting protein. Additionally, molecular glue generally exhibits lower molecular weights, making them inherently more compliant with Lipinski's Rule‐of‐Five [10, 11]. These advantages establish molecular glue as an efficient strategy for the inactivation of therapeutic targets intractable by conventional pharmacological means [12]. Mechanistically, characterized molecular glues bind E3 ubiquitin ligase substrate receptors to recruit target proteins for proteasomal degradation. Previous study demonstrated that thalidomide analogues recruit zinc‐finger transcriptions factors and other targets to CRBN, the substrate receptor of cullin‐RING E3 ubiquitin ligase CUL4A/B‐RBX1‐DDB1‐CRBN (CRL4^CRBN^) [13]. Similarly, aryl sulphonamides deplete the essential RNA‐binding protein RBM39 by engaging DCAF15, the substrate receptor of CRL4^DCAF15^ [14]. Molecular glue may also stabilize ternary complexes by facilitating interactions between substrates and adaptor proteins like DDB1 [15].

Cullin‐RING ‐E3 ubiquitin ligase complex (CRLs) is central to molecular glue mediated protein degradation. Among these, CRL4 complex employs a similar substrate‐adaptor mechanism to target various proteins for ubiquitin dependent proteolysis, and DDB1 has been proposed to be adaptor protein in CRL4 complex for substrate‐targeting [16]. The complex achieves substrate specificity by engaging DDB1‐CUL4‐associated factors (DCAFs) and select WD40 repeat domain (WDR) protein family members as substrate receptors [17, 18]. These receptors share structural hallmarks—WD40 *β*‐propeller domains and helix‐loop‐helix motifs—that facilitate DDB1 binding. CUL4 comprises two conserved paralogs, CUL4A and CUL4B. It has been reported that CUL4A and CUL4B equally interact with most previously identified DCAFs, while a small subset was found to preferentially bind CRL4A, for example, CRBN prefers to assemble with CRL4A [19]. Currently, rational design strategies have been broadly applied for CRBN‐directed molecular glues [20, 21], yet analogous approaches targeting other DCAFs and WDR family members remain underdeveloped. Additionally, while molecular glues hold immense therapeutic promise, their discovery remains largely serendipitous, and targeting extracellular or transmembrane protein remains challenging [22].

In this study, we demonstrate that halofuginone (HF), a derivative of alkaloids originally isolated from the plant halofuginone, suppressed tumor cells progression through the mechanism by which it functions as a new molecular glue. Identified through high throughput screening from a natural chemical library, HF found to possess potent cytotoxicity to ESCC cells both in vitro and in vivo. PEGylation of HF (PEG‐HF) preserved efficacy while markedly attenuating systemic toxicity in tumor‐bearing mice. HF functions as a ternary complex stabilizer, binding integrin *β*4 and recruiting it to the substrate receptor WDR18 of E3 ubiquitin ligase CRL4B complex, thereby triggering the degradation of integrin *β*4. Additionally, we also revealed that high expression of integrin *β*4 is associated with aggressive phenotype of tumor cell, and a worse prognosis of ESCC. Furthermore, we demonstrated that HF administration as well as integrin *β*4 depletion led to apoptosis and the activation of Hippo signaling.

## 2 Results

### 2.1 HF Exhibits High Inhibitory Effect on ESCC Cells

**FIGURE 1:**
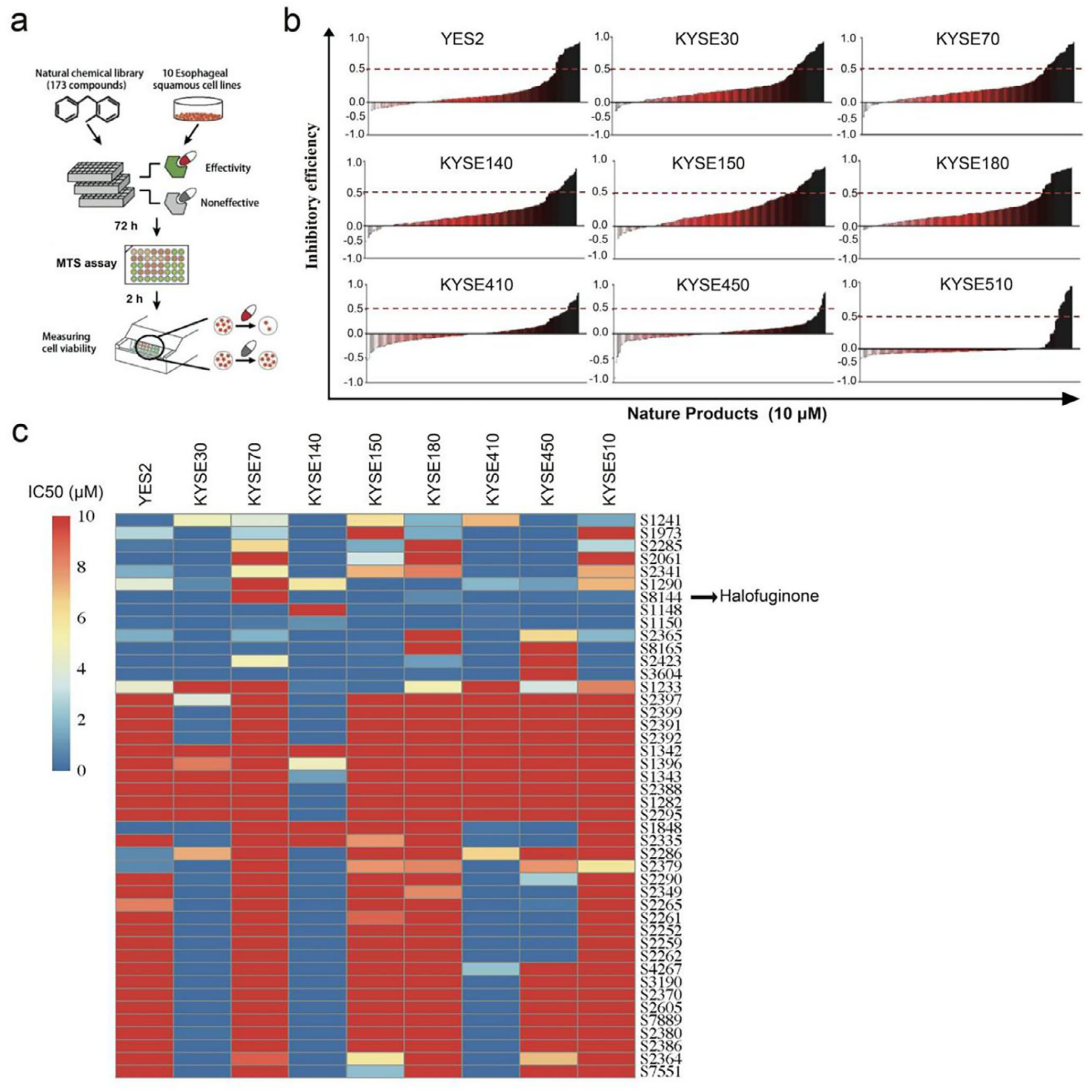
High throughput screening of candidate natural products. (a) Schematic diagram of drug screening process. (b) 10 µM of diluted 173 molecules in a natural products library were added to nine ESCC cell lines, 72 hours later, MTS assay was performed to screen candidate molecules with an inhibitory rate larger than 50%. A series dilution (0, 0.001, 0.01, 0.05, 0.1, 0.5, 1.0, 2.5, 5, 10 µM) of 44 molecules that obtained in Figure 1b were added to nine ESCC cell lines for a second screen, 72 hours later, IC_50_ values were calculated according to the MTS results. All data are presented as mean ± s.d. Statistical significance was determined by a two‐tailed Student's t‐test. *P < 0.05, **P < 0.01, ***P < 0.001.

To identify cytotoxic compounds against esophageal squamous cell carcinoma (ESCC), we performed high‐throughput screening of a 173‐natural compound library across nine ESCC cell lines using MTS viability assays (Figure 1a). Primary screening identified 44 compounds exhibiting >50% growth inhibition in all cell lines (Figure 1b) Dose‐response validation (0, 0.001, 0.01, 0.05, 0.1, 0.5, 1.0, 2.5, 5, 10 µM) revealed HF as the most potent candidate, with the IC50 values ranging from 0.2492 to 24.77 µM (Figure 1c; Figure S1a, b). Therefore, we put our focus on HF, aimed to dissect detailed mechanism of its inhibitory effect on ESCC cell.

### 2.2 HF Inhibits Aggressive Phenotype of ESCC Cells Both In Vitro and In Vivo

**FIGURE 2:**
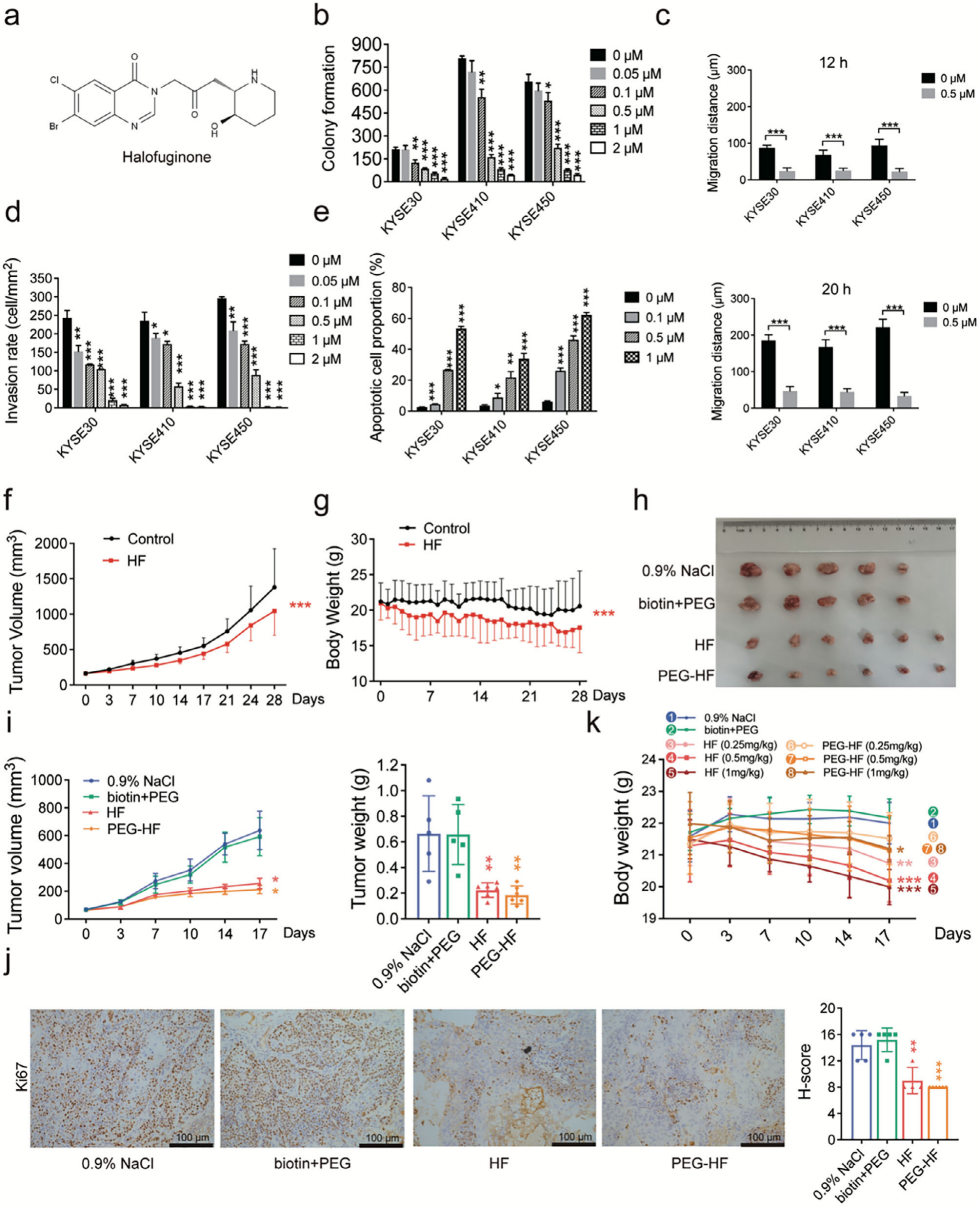
HF exhibits inhibitory effect to ESCC cells. (a) The chemical formula of HF. (b‐e). HF administration inhibits ESCC aggressive phenotypes (Colony formation in Figure 2b, Migration ability in Figure 2c, Invasion ability in Figure 2d, Apoptosis in Figure 2e) on a dose‐dependent manner in KYSE30, KYSE410 and KYSE450 cells. (f) HF administration suppresses tumor growth in eight PDX model (n = 8). (g) HF exposure exhibits severe side effect on tumor bearing PDX model weight (n = 8). (h, i) Macroscopic appearance of the xenograft tumors in nude mice after intragastric administration of 0.9% NaCl, biotin plus PEG, HF and PEG‐HF respectively, as well as tumor volume and tumor weight in each group (n = 6). (j) IHC that with Ki67 staining of different xenografts, the scale bar is 100 µm, as well as the H‐Score analysis (n = 6). (k) Mouse body weight analysis to test whether PEG‐HF administration will improve the side effect compared to HF exposure (n = 6). All data are presented as mean ± s.d. Statistical significance was determined by a two‐tailed Student's t‐test. *P < 0.05, **P < 0.01, ***P < 0.001.

HF, a bioactive alkaloid derived from the *Dichroa febrifuga* (Figure 2a), is known for its broad‐spectrum antiparasitic drug for animals. Recent studies revealed its therapeutic potential in autoimmune disease and cancers [23], with inhibitory effect on angiogenesis, metastasis, and cell proliferation of tumor cells [24]. To comprehensively characterize the anti‐tumor activity of HF, we started by conducting a dose‐response of HF exposure to the ESCC cell lines KYSE30, KYSE410 and KYSE450. As anticipated, HF treatment exhibited dose‐dependent inhibitory effect to ESCC cell lines, with significantly decreased the colony formation (Figure 2b; Figure S2a), markedly attenuated the migration distance (Figure 2c; Figure S2b), decreased the invasion ability (Figure 2d; Figure S2c), and greatly induced high percentage of apoptosis (Figure 2e; Figure S2d). We next evaluated the therapeutic efficacy of HF using the patient‐derived xenograft (PDX) models (Figure S2e). Daily intragastric HF administration (0.5 mg/kg) significantly suppressed tumor growth (Figure 2f) but caused substantial body weight loss (Figure 2g), suggesting a potential side effect of HF administration that necessitates structural optimization.

**FIGURE 3:**
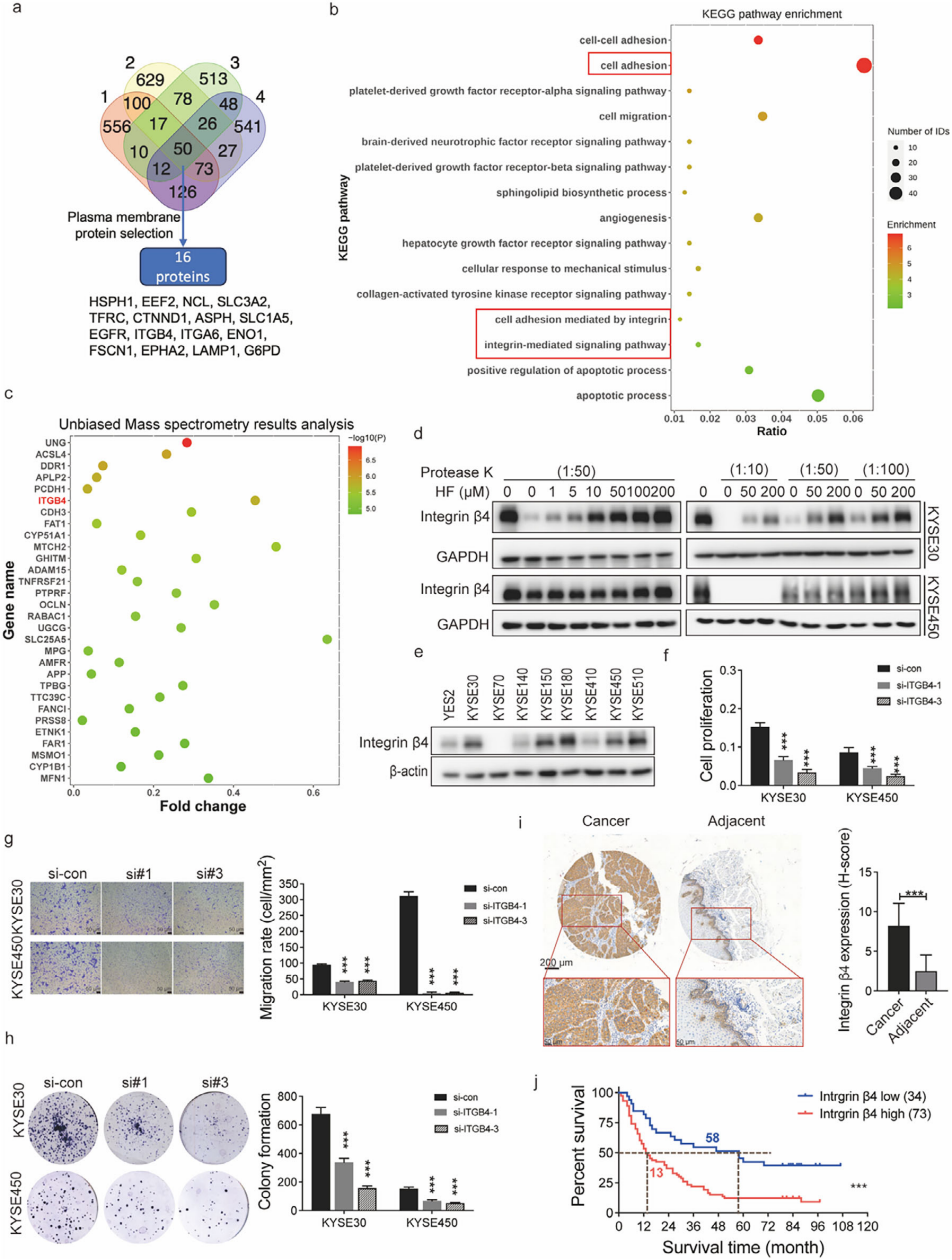
Integrin *β*4 is a promising target for HF. (a) Analysis pipeline was performed to identify proteins that interact with HF: (1) Screening 16 plasma membrane proteins in the overlapped 50 proteins from the four bands; (2) Selecting proteins with high quantity of unique peptides and MS score, and integrin *β*4 was identified to bind with HF in mass spectrometry results. (b) Proteomic analysis was conducted to assess the global downstream changes induced by HF treatment in KYSE30 cell, accompanied by KEGG pathway analysis. (c) Properties of the top 30 proteins in global downstream changes induced by HF treatment in KYSE30 cell. (d) DARTs‐western blot assay was performed to determine whether HF protects integrin *β*4 from degradation after protease K exposure both in KYSE30 and KYSE450 cell. (e) Western blot to reveal the expression profile of integrin *β*4 in ESCC cell lines. (f‐h) MTS assay to determine the cell viability (Figure 3f), invasion ability (Figure 3g) and colony formation ability (Figure 3h) after knockdown of integrin *β*4. (i) IHC that with integrin *β*4 staining in a tissue microarray containing 107 dots of ESCC tissue and 72 dots of adjacent tissues, the scale bar is 200 µm. (j) Kaplan‐Meier survival analysis of ESCC patients’ overall survival based on the abundance of integrin *β*4. All data are presented as mean ± s.d. Statistical significance was determined by a two‐tailed Student's t‐test. *P < 0.05, **P < 0.01, ***P < 0.001.

PEGylation—covalent attachment of polyethylene glycol (PEG) to therapeutics—is a clinically validated strategy for improving pharmacokinetic profiles and therapeutic indices. More than 30 PEGylated drugs have been used in the clinical practice, and many investigational PEGylated agents are undergoing clinical trials [25]. To mitigate HF‐associated toxicity while preserving efficacy, we synthesized PEG‐HF through covalent PEG‐biotin conjugation (Figure S2f, g). Consistently, PEG‐HF were shown to inhibit ESCC cell growth in vitro (Figure S2h). Daily oral gavage administration was performed in xenograft tumor‐bearing nude mice using various concentrations of HF, PEG‐HF, 0.9% NaCl, and biotin‐PEG combination. The results demonstrated that both HF and PEG‐HF treatments exhibited significant antitumor effects, as evidenced by: (1) visibly smaller tumor sizes (Figure 2h), (2) reduced tumor volume and weight measurements (Figure 2i), and (3) decreased Ki67 proliferation marker expression (Figure 2j). Notably, while these therapeutic interventions showed promising antitumor efficacy, the body weight of tumor‐bearing mice displayed significant variations among treatment groups. As indicated in Figure 2k, compared to the control group, low dose (0.25 mg/kg) of HF administration would lead to a markedly loss of body weight, while medium dose (0.5 mg/kg) of PEG‐HF exposure exhibited no significant change in body weight. Although high dose (1 mg/kg) of PEG‐HF resulted in weight loss in mice, their body weight was still higher than that of mice exposed to low dose of HF (0.25 mg/kg). We also checked the serum biochemical index of those tumor bearing mice, including total protein, albumin, creatinine, urea, aspertate aminotransferase, alanine transaminase, direct bilirubin and γ‐glutamyltransferase (Figure S3a), confirmed markedly attenuated systemic toxicity with of PEGylated HF. Additionally, histopathological analysis revealed that neither HF nor PEG‐HF caused damage to the morphology of mouse liver and kidney tissues (Figure S3b). Collectively, HF inhibits the aggressive phenotype of ESCC cell both in vitro and in vivo but exhibits side effects in vivo experiments, while PEGylated HF extends to be equally functional with fewer side effects.

### 2.3 Integrin *β*4 is a Promising Target of HF

Given the growth and invasion inhibitory effect of HF in ESCC cells, we sought to identify the potential targets of HF. Drug affinity responsive target stability (DARTs), which takes advantage of a reduction in the protease susceptibility of the target protein upon drug binding, has been widely used to identify small molecule‐protein interaction [26]. We performed DARTs assay by incubating different concentrations of HF and KYSE30 cell lysate, and the subsequent protease K addition. SDS‐PAGE, silver staining and the following mass spectrometry assay were used to detect HF binding proteins (Figure S4a). By integrating data from the Human Protein Atlas database [27] (proteinatlas.org), we identified 16 membrane‐associated proteins for their high quantity of unique peptides and MS score in the mass spectrometry data (Figure 3a). Furthermore, proteomic analysis was performed to investigate the comprehensive downstream effects of HF treatment. As illustrated in Figure 3b, pathway analysis identified significant enrichment in integrin‐mediated cell adhesion and signaling pathways. Critically, this was accompanied by a substantial decrease in integrin *β*4 abundance post‐HF administration (Figure 3c). We repeated the DARTs assay, and confirmed dose‐dependent stabilization of integrin *β*4 against proteolysis with HF treatment (Figure 3d). Collectively, these data suggest the potential interaction between HF and integrin *β*4, accompanied by a reduction in integrin *β*4 levels due to HF exposure.

We observed that integrin *β*4 heterogeneously expressed in ESCC cell lines (Figure 3e). Knockdown of integrin *β*4 (Figure S4b) markedly led to cell growth inhibition (Figure 3f), decreased invasion ability (Figure 3g), and declined colony formation ability (Figure 3h). To explore the expression characteristics and prognostic relevance of integrin *β*4 clinically, we examined the expression level of integrin *β*4 in tissue microarrays that contain 107 ESCC tissue and 72 adjacent tissues. Integrin *β*4 displayed significantly higher expression level in ESCC tissues compared to the matched adjacent tissue (Figure 3i), and higher integrin *β*4 expression was associated with lymph node metastasis (Table S2) and a worse survival for patients with ESCC (Figure 3j). Additionally, Cox regression analysis showed that high expression of integrin *β*4 could be severed as an independent influencing factor for survival prognosis (Figure S4c). Consistent with these data, our analysis of integrin *β*4 expression in GEPIA [28] and KM‐plotter database [29, 30] also uncovered that higher integrin *β*4 expression was associated with a worse survival across multiple solid tumors (Figure S4d, e), suggesting a pivotal role of integrin *β*4 in promoting tumor malignant process.

### 2.4 HF Directly Binds Integrin *β*4 and Mediates Its Degradation

**FIGURE 4:**
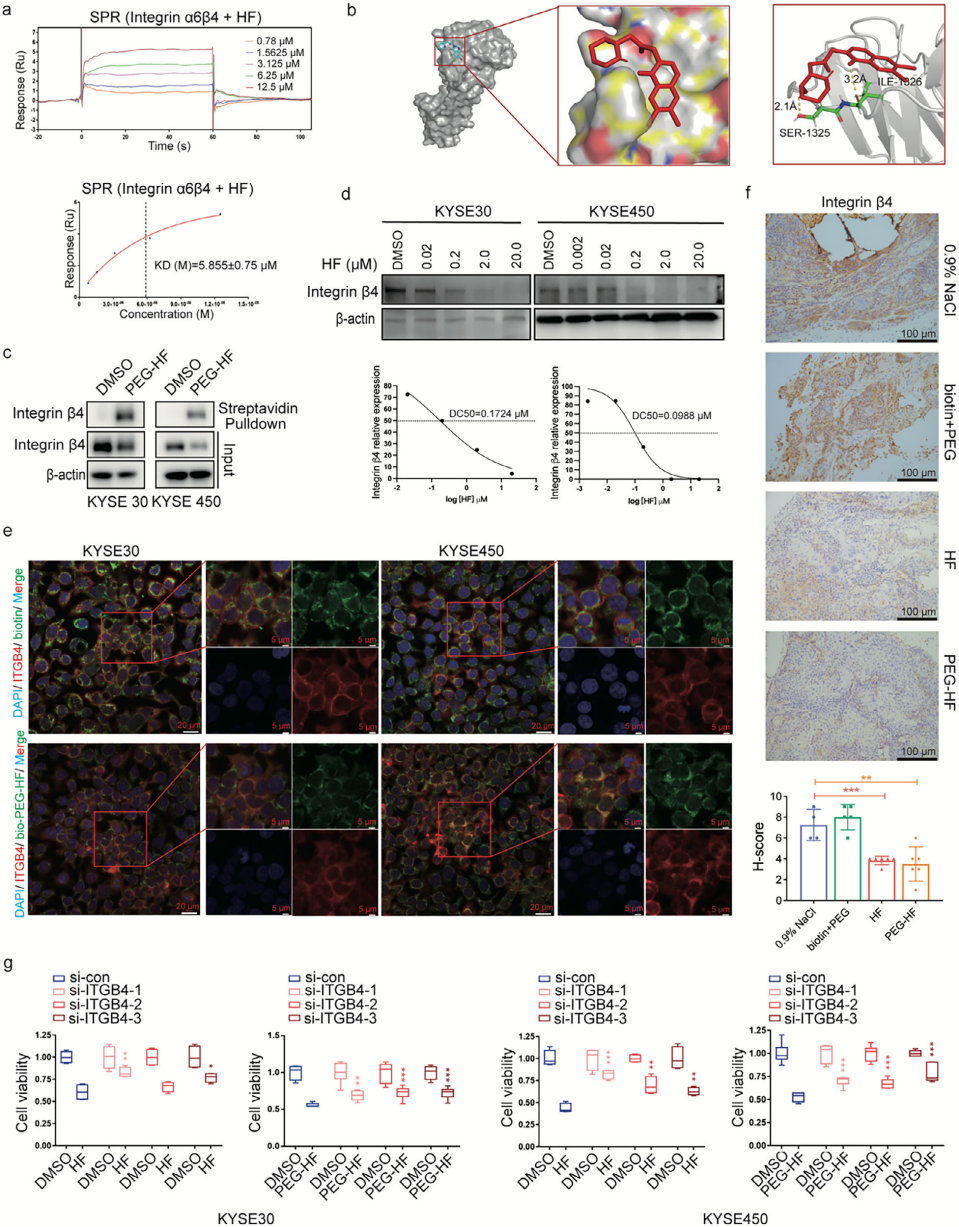
HF binds to integrin *β*4 and mediates its degradation. (a) SPR assay was performed to test the binding ability of HF to integrin *β*4. (b) Molecular docking analysis of the binding ability between HF and integrin *β*4. (c) Streptavidin‐pulldown and western blot assay were executed to test the binding ability of HF to integrin *β*4. (d) Western blot to determine the expression level of integrin *β*4 upon different concentration of HF exposure and the following DC50 and Dmax calculation. (e) Confocal assay to test the co‐localization of PEG‐HF and integrin *β*4 in KYSE30 and KYSE450, the scale bar is 20 µm. (f) IHC that with integrin *β*4 staining in different xenografts, the scale bar is 200 µm (n = 6). (g) MTS experiments were executed to test the role of integrin *β*4 abundance in the susceptibility of ESCC cell line to HF. All data are presented as mean ± s.d. Statistical significance was determined by a two‐tailed Student's t‐test. *P < 0.05, **P < 0.01, ***P < 0.001.

To test whether HF can directly bind integrin *β*4, we performed a surface plasmon resonance (SPR) assay. Briefly, purified integrin *β*4 was immobilized on a sensor chip, then different concentrations of HF were infused and went through the surface of the microarray, the binding affinity indexes of integrin *β*4 and HF were recorded. SPR results showed that the dissociation constant (KD) index of integrin *β*4 and HF was 5.855 ± 0.75 µM, indicating a moderate interaction (Figure 4a). Additionally, molecular docking (AutoDock) [31] identified a binding pocket in integrin *β*4. As shown in Figure 4b, HF formed two hydrogen bonds with the serine residue at position 1325 and the isoleucine residue at position 1326 of integrin *β*4, with distances of 2.1Å and 3.2Å, respectively. The <5 Å spacing between these bonds satisfies criteria for stable physical interactions. Consistently, streptavidin pulldown assay also demonstrated the interaction of integrin *β*4 and HF (Figure 4c). In addition, we observed that HF exposure induced dose‐dependent degradation of integrin *β*4 with DC_50_ values of 0.1724 µM in KYSE30 and 0.0988 µM in KYSE450 cell, and the maximum degradation (Dmax) of integrin *β*4 in both cells exceeded 96% (Figure 4d). Confocal detection also confirmed the co‐localization of integrin *β*4 and HF and a blurring cell contour after HF exposure (Figure 4e), further supporting the interaction between integrin *β*4 and HF and HF induced degradation of membrane localized integrin *β*4. Similarly, integrin *β*4 immunohistochemical staining in xenograft tumors demonstrated that HF treatment induced degradation of integrin *β*4 (Figure 4f). The siRNA‐mediated *ITGB4* knockdown in KYSE30/KYSE450 cells significantly reduced HF sensitivity (Figure 4g), indicating that HF‐induced cytotoxicity requires integrin *β*4. Collectively, these data demonstrate HF binds integrin *β*4, induces its degradation, and exerts cytotoxic effects primarily through integrin *β*4 depletion.

### 2.5 HF Induces Proteasomal Degradation of Integrin *β*4 by Conferring Glue Activity

**FIGURE 5:**
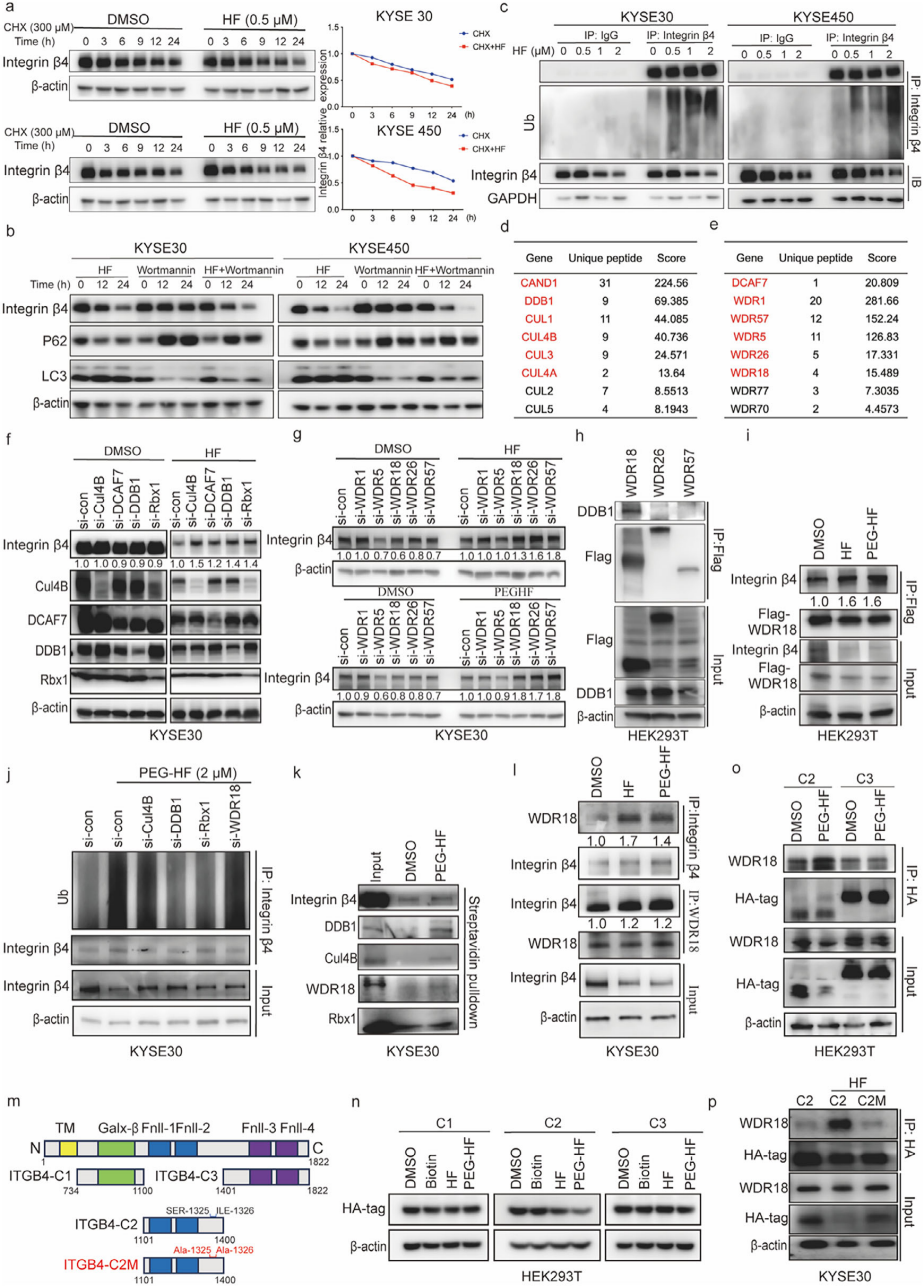
HF promotes proteasomal degradation of integrin *β*4 by conferring glue activity. (a) Western blot to determine the degradation rates of integrin *β*4 by HF exposure (0.5 µM) after adding protein synthesis inhibitor CHX (300 µM) in KYSE30 and KYSE450 cell. (b) Western blot was performed to find the pathway of HF induced integrin *β*4 degradation in KYSE30 and KYSE450 cell, wortmannin was use to inhibit autophagy. (c) Western blot to determine the ubiquitination of integrin *β*4 upon HF administration in ESCC cells, briefly, KYSE30 and KYSE450 cell were exposed to different concentrations of HF (0–2 µM), then, total cell extracts were subjected to IP using an anti‐integrin *β*4 antibody, ubiquitin was detected in the immunoprecipitates by western blot. (d, e) Cullin associated proteins and potential substrate receptors with the highest scores in mass spectrometry result. (f, g) Western blot was performed to test whether knockdown of these proteins would result in integrin *β*4 abundance change within or without HF exposure. (h) IP‐western blot was performed to identify which substrate receptors could bind to DDB1. Briefly, the reconstituted plasmids containing a 3×flag tag on the N terminal of WDR18, WDR26 and WDR57, were transfected to HEK293T cell, then, total cell extracts were subjected to IP using Anti‐flag magnetic beads, flag tag and DDB1 was detected in the immunoprecipitates by western blot. (i) Western blot analysis of integrin *β*4 abundance in the immunoprecipitates enriched by anti‐flag magnetic beads in HEK293T cell that transfected with Flag‐tagged WDR18. (j) Western blot analysis of ubiquitinzed integrin *β*4 after knockdown of CRL4B^WDR18^ components. (k) Streptavidin‐pulldown and western blot were performed to determine the binding ability of HF to CRL4^WDR18^ E3 ligase components. (l) Co‐IP and western blot analysis of the binding affinity between integrin *β*4 and WDR18 upon HF and PEG‐HF administration in KYSE30 cell. (m) Construction of truncated ITGB4 plasmids and the mutant plasmid. (n) HEK293T cells were transfected with ITGB4‐C1, ITGB4‐C2 or ITGB4‐C3, and then the transfected cells were exposed to DMSO, biotin, HF and PEG‐HF, respectively, HA tag was detected by western blot. (o) HEK293T cells were transfected with ITGB4‐C2 or ITGB4‐C3 for 24 h, then the transfected cells were treated with PEG‐HF, total cell extracts were subjected to IP using an anti‐HA antibody, WDR18 and HA tag were detected in the immunoprecipitates by western blot. (p) IP‐western blot was performed to test whether the key sites mutation in ITGB4‐C2 would disrupt its binding to HF. Briefly, HEK293T cells were transferred with ITGB4‐C2 and ITGB4‐C2M, respectively. The transferred cells were treated with HF overnight. Total cell extracts were subjected to IP using an anti‐HA antibody, WDR18 and HA tag were detected in the immunoprecipitates by western blot.

We further dissected the underlying mechanism of HF‐induced integrin *β*4 degradation. KYSE30 and KYSE450 cells were treated with CHX to block the synthesis of integrin *β*4, which lead to an increased degradation rates of integrin *β*4 within HF administration (Figure 5a), indicating HF mediated the post‐translational change of integrin *β*4. Proteins degradation pathways can be divided into two parts, proteasomal and lysosomal pathways [32]. We thus sought to investigate whether HF‐induced integrin *β*4 degradation depends on the proteasomal pathway or the lysosome pathway. Wortmannin, an inhibitor of autophagy formation, successfully boosted p62 expression and decreased the expression levels of LC3 I/II (Figure 5b), confirming the inhibition of autophagy formation, however, autophagy inhibition did not affect the abundance of integrin *β*4 (Figure 5b), while proteasome inhibitor Mg132 administration significantly attenuated the HF‐induced integrin *β*4 degradation (Figure S5a). In addition, immunoprecipitation results showed that HF administration promoted the ubiquitination of integrin *β*4 on a dose‐dependent manner in ESCC cells (Figure 5c). Collectively, these findings suggest HF mediates the integrin *β*4 degradation through proteasomal pathway.

Molecular glues represent an emerging therapeutic strategy that stabilizes protein‐protein interactions, often leading to target degradation when engaging E3 ubiquitin ligases [15]. We speculated that HF may act as a molecular glue that induced high affinity binding of integrin *β*4 and E3 ubiquitin ligase components. In the mass spectrometry data of potential HF‐bound proteins, we identified a series of cullin associated proteins, including cullin associated proteins CAND1, DDB1, Cul1, Cul4B, Cul3, Cul4A, and substrate receptors DCAF7, WDR1, WDR5, WDR18, WDR26 and WDR57 (Figure 5d, e). Functional screening via siRNA knockdown demonstrated that Cul4B, DDB1, and Rbx1 (but not Cul1/3/4A or CAND1) were essential for HF‐induced integrin *β*4 degradation (Figure 5f; Figure S5b). Cullin‐RING ligases engage their substrates through substrate receptors, with DDB1 serves as an adaptor protein that binds such receptors (also known as DDB1‐Cul4‐associated‐factors, DCAFs) to recruit them to the Cul4‐Rbx1 ligase core [33]. DCAF7 was identified in our mass spectrometry data, however, knockdown of *DCAF7* failed to attenuate integrin *β*4 degradation induced by HF administration (Figure 5f). In addition to DCAF family members, WDR family members were also reported to be the substrate receptor of CRL4B complex [18, 34]. We found that downregulation of WDR18, WDR26 and WDR57 via siRNAs significantly inhibited the degradation of integrin *β*4 upon HF or PEG‐HF administration (Figure 5g; Figure S5c). Subsequently, co‐IP assay was used to identify the substrate receptor of integrin *β*4 in the CRL4B complex. As shown in Figure 5h, WDR18 was the only protein that interacts with DDB1. Additionally, HF and PEG‐HF administration increased the binding intensity between Flag‐tagged WDR18 and integrin *β*4 in HEK293T cell (Figure 5i), suggesting WDR18 acts as the receptor of integrin *β*4 in the CRL4B complex. We also observed that knockdown of CRL4B^WDR18^ components significantly impaired PEG‐HF induced integrin *β*4 ubiquitination (Figure 5j). Consistently, the following streptavidin pulldown assay showed that PEG‐HF could bind to the CRL4B related proteins (Figure 5k). Furthermore, knockdown of Cul4B largely inhibited the degradation rate of integrin *β*4 upon PEG‐HF and the protein synthesis inhibitor CHX exposure (Figure S5d). Moreover, co‐IP assays demonstrated HF and PEG‐HF administration significantly enhanced the interactions between integrin *β*4 and WDR18 (Figure 5l). Collectively, HF functions as a molecular glue to promote integrin *β*4 interaction with WDR18, and mediates its degradation through CRL4B^WDR18^ E3 ligase.

Integrin *β*4 is well known for its large cytoplasmic domain with more than 1,000 amino acids, which was artificially divided to three parts according to its structure and function, namely, domain closed to membrane (termed C1 from 734 aa to 1100 aa), the first pair of FNII (termed C2 from 1101 aa to 1400 aa), the second pair of FNII (termed C3 from 1401 aa to 1822 aa). C2 is well known for its critical role in hemidesmosome formation, while C3 is suggested to link to actin cytoskeleton [6]. To map the functional domains of integrin *β*4 critical for HF's effects, we expressed HA‐tagged truncation constructs (C1, C2, C3) in HEK293T cells (Figure 5m). Strikingly, HF and PEG‐HF treatment selectively depleted the C2 domain (Figure 5n), consistent with our docking data showing HF binding to Ser1325 and Ile1326 within this region (Figure 4b). In Addition, Co‐IP assays revealed that PEG‐HF specifically enhanced the interaction between C2 (but not C3) and WDR18 (Figure 5o), confirming domain‐selective engagement. To determine the importance of Ser1325 and Ile1326 in integrin *β*4 for its binding to HF, we constructed a C2 mutant plasmid by mutating both residues to alanine (Figure 5m). As indicated in Figure 5p, Ser1325 and Ile1326 mutation would abolish the HF‐induced C2‐ITGB4 degradation. Co‐IP results indicated that HF specifically interacts with C2‐ITGB4 but not the C2‐ITGB4 mutant protein, highlighting the critical role of these two sites in HF binding (Figure 5p). Collectively, these results suggest that HF binds the first pair of FNII motif (C2) of integrin *β*4 and triggers the proteasomal degradation of integrin *β*4 through the CRL4B^WDR18^ involved ubiquitin proteasomal pathway.

### 2.6 HF Promotes Apoptosis of Tumor Cell and Activates Hippo Signaling

**FIGURE 6:**
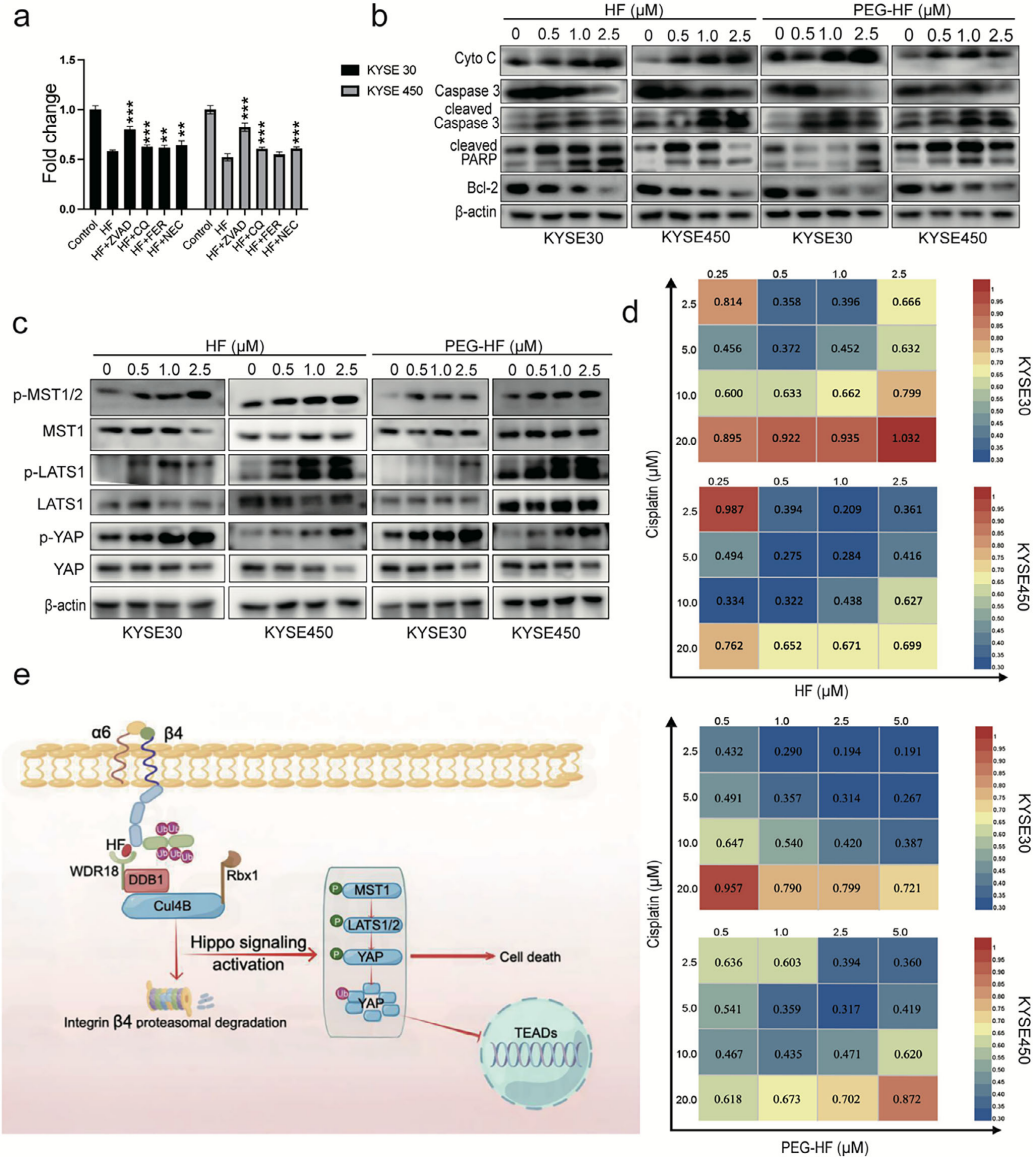
HF promotes apoptosis of tumor cell. (a) Chloroquine as an autophagy inhibitor (50 µM), ZVAD‐fmk as an apoptosis inhibitor (20 µM), Necrostatin‐1 as a necrosis inhibitor (30 µM), Ferrostatin‐1 as a ferroptosis inhibitor (1 µM) were separately added to HF‐exposed (0.5 µM) KYSE30 and KYSE450 cell for 72 h, cell viability was detected by MTS. (b) Apoptosis associated proteins were detected upon HF or PEG‐HF exposure by western blot in ESCC cells. (c) Hippo signaling pathway related proteins were detected after HF or PEG‐HF treatment by western blot in ESCC cells. (d) Different concentrations of cisplatin (2.5, 5, 10, 20 µM) and HF (0.25, 0.5, 1, 2.5 µM) or PEG‐HF (0.5, 1, 2.5, 5 µM) were co‐added to KYSE30 and KYSE450 cell for 72 h, respectively, MTS assay were performed to determine the cell viability, the Combination Index (CI) was calculated in CalcuSyn software, CI from 0.2 to 0.4 refers a strong synergistic effect, CI from 0.4 to 0.6 indicates a high synergistic effect, CI from 0.6 to 0.8 denotes a moderate synergistic effect, while CI from 0.8 to 0.9 suggests a low synergistic effect. (e) The working model by which HF promotes apoptosis by acting as a molecular glue degrader to promote integrin *β*4 degradation. All data are presented as mean ± s.d. Statistical significance was determined by a two‐tailed Student's t‐test. *P < 0.05, **P < 0.01, ***P < 0.001.

Given the growth inhibitory effect of HF and PEG‐HF, we sought to Figure out the cell fate upon HF or PEG‐HF exposure. Chloroquine, ZVAD‐fmk, Necrostatin‐1 and Ferrostatin‐1, which were respectively known for their autophagy, apoptosis, necrosis, and ferroptosis inhibitory effect, were added to ESCC cell lines to detect the rescue effect of HF induced tumor cell cytotoxicity by MTS. The results demonstrated that Chloroquine, Necrostatin‐1 and Ferrostatin‐1 could partially rescue the HF‐induced cytotoxicity, while ZVAD‐fmk administration significantly improved the cell viability compared to cell treated with HF alone (Figure 6a). In consistency with the MTS assay, western blot results showed that HF exposure led to the increased protein levels of Cyto C, cleaved caspase‐3, cleaved PARP, while decreased Bcl‐2 expression on a dosage‐dependent manner (Figure 6b). Together, these results suggested that HF exposure induced cell death relied mainly on apoptosis. As mentioned in Figure 5, HF binds the first pair of FNII motif (C2) of integrin *β*4 and mediates CRL4B^WDR18^ involved proteasomal degradation. Considering epithelial cells highly rely on appropriate cell‐matrix interaction for cell anchorage and survival, as well as the pivotal role of integrin *β*4 in hemidesmosome formation and subsequent extracellular matrix adhesion [5], we proposed that HF promoted apoptosis by increasing integrin *β*4 degradation might involve decrease of cell and extracellular matrix adhesion. In the context of signaling that cell adhesion involved cell death, Hippo pathway exhibits more active [35, 36]. We then detected the dynamic change of Hippo pathway, and found that both HF and PEG‐HF administration promoted phosphorylation of MST1, LATS1 and YAP on a dose‐dependent manner (Figure 6c). To assess the dependence of the Hippo pathway on HF‐induced cell death, we administered HF to YAP‐overexpressing ESCC cells and calculated the IC_50_ of HF in these cells. The cell viability results demonstrated that YAP overexpression significantly attenuated the cytotoxicity of HF to ESCC cells, highlighting the crucial role of Hippo pathway in determining HF‐induced cell death (Figure S5e). Collectively, we demonstrated that HF exposure stimulates Hippo signaling pathway and results in apoptosis in the end.

Given cisplatin's widespread use in solid tumor therapy, we investigated whether HF or PEG‐HF could potentiate its activity in ESCC. KYSE30 and KYSE450 cells were treated with cisplatin (2.5–20 µM) in combination with HF (0.25–2.5 µM) or PEG‐HF (0.5–5 µM) for 72 hours. The Combination Index (CI) values were subsequently calculated using CalcuSyn software, where a lower CI value indicates a stronger synergistic effect. The results showed that cisplatin combining with HF or PEG‐HF exposure exhibited a synergistic effect in inhibiting viability of ESCC cells, among which 0.5∼1 µM HF and 2.5∼10 µM cisplatin combination exhibited the highest synergistic effect, while 2.5∼5 µM PEG‐HF combined with 2.5∼5 µM cisplatin presented the best co‐inhibitory efficacy (Figure 6d). Taken together, HF and PEG‐HF administration can promote apoptosis, and have high clinical application potential.

## 3 Discussion

Integrins are vital surface adhesion receptors that mediate interactions of extracellular matrix and cells and crucial to a wide range of biological functions. Several marketed treatments targeted integrins *α*IIb*β*3, *α*4*β*7, *α*4*β*1 and *α*L*β*2 have been successfully used for many clinical diseases [4]. Integrin *β*4, as a critical structural role in the hemidesmosome of epithelial cells, is required for the regulation of keratinocyte polarity and motility [6]. The critical role of biological events and clinical relevance have made integrin *β*4 a potential therapeutic target. However, integrin *β*4 targeting approaches have not been well explored. Through high throughput screening of a natural product library, we identified HF as a candidate small molecule with high cell growth inhibitory efficiency. Mechanically, HF, functions as a molecular glue, promotes integrin *β*4 degradation by increasing the interaction of integrin *β*4 and CRL4B^WDR18^ E3 ligase, and Hippo signaling pathway activation and the following apoptosis (Figure 6e).

Growing evidence suggests that integrin *β*4 is present at high levels in various cancer types, and high expression of integrin *β*4 correlates with poor prognosis, including lung cancer, breast cancer, pancreatic cancer, gastric cancer and cervical cancer [7, 8, 9]. Here, we demonstrated integrin *β*4 displayed high expression in ESCC and high integrin *β*4 abundance was correlated with worse prognosis (Figure 3j). Given the critical role of integrin *β*4 in tumor cell growth and metastasis, several approaches have been trying to target integrin *β*4 for clinical treatment. For example, Shasha Ruan et al. reported that immunologic strategies targeting integrin *β*4 represented a novel approach to target cancer stem cell [37]. Shuangmei Tong et al. demonstrated that the IDH1‐mutant metabolite D‐2HG suppressed integrin *β*4 expression, and down‐regulated the phosphorylation levels of PI3K and AKT, ultimately inhibiting cell proliferation via promoting apoptosis, thereby improving glioma prognosis [38]. The CSTONE PHARMACEUTICALS (a biopharmaceutical company) had developed one kind of antibody conjugated drug (ADC) by targeting integrin *β*4 for the clinical use of various solid tumors. Through high throughput screening, we identified a natural product HF that could induce proteasomal degradation of integrin *β*4 via a molecular glue‐like mechanism. Specifically, we demonstrated the direct binding of HF to the C2 motif of integrin *β*4 and the subsequent HF‐induced degradation of this motif, which is known to be essential for hemidesmosome formation [5, 6]. Therefore, we speculate that HF treatment leads to integrin *β*4 C2 motif degradation, resulting in failed hemidesmosome formation and subsequent cell death. This finding addresses a critical gap in targeted protein degradation research, where current molecular glue strategies predominantly focus on intracellular proteins while neglecting transmembrane targets [39, 40]. This work provides the experimental evidence that molecular glue compounds can effectively target transmembrane proteins for degradation, broadening avenues for developing membrane protein degraders.

Accumulating evidences also showed that the natural products are a primary source of anticancer drugs and have played important roles in the development of targeted protein degradation (TPD) [10, 41, 42]. Besides the HF‐induced integrin *β*4 degradation, both in vitro or in vivo experiments indicated that HF exposure markedly inhibited the malignant phenotypes of tumor cell, and HF treatment could enhance the therapeutic sensitivity of cisplatin in tumor cell, suggesting a promising of HF or HF combination therapy in clinical applications. However, the side or toxic effects of HF might greatly impair its potential for clinical application. We demonstrated that PEGylated HF extended to be similarly functional to HF with lower side effect in body weight loss, liver and kidney toxicity in the tumor bearing mouse model. Although in vivo experiments need to be conducted to demonstrated the synergistic effect of HF and PEG‐HF combined with cisplatin, cell experiments have already confirmed that HF and PEG‐HF combined cisplatin can exhibit a high synergistic effect. However, we cannot neglect the declined inhibitory efficiency of PEG‐HF compared to HF (Figure S2h). A previously published study unveiled that CDK inhibitor CR8 acted as a molecular glue degrader that depleted Cyclin K through its hydrophobic phenylpyridine ring system [15]. While the other CDKs inhibitor DRF053 that carries a differently linked phenylpyridine ring system, and the parent compound of CR8 Roscovitine which lacks the 2‐pyridyl substituent but retains the phenyl ring proximal to Arg928, showed less molecular glue effects, suggesting chemical alteration of surface exposed moieties can confer gain‐of‐function glue properties to an inhibitor [15]. Therefore, for higher glue like and inhibitory efficiency, more rational design or chemical biology‐based modification of HF are needed in the future study.

Previous studies have reported several molecular glues, for example, thalidomide analogues acts to recruit zinc‐finger transcription factors and other targets to CRL4^CRBN^, aryl sulphonamides degrades RBM39 by engaging CRL4^DCAF15^, HQ461 promotes CDK12‐DDB1 interaction to trigger cyclin K degradation, CDK inhibitor CR8 functions as a molecular glue degrader to deplete cyclin K. Additionally, a recent study revealed that intramolecular bivalent glues simultaneously engage and connect two adjacent domains of BRD4 in cis, and this conformational change glues BRD4 to DCAF11 or DCAF16 [43], indicating the diversity of mechanisms involved in the molecular glue degraders induced targeted proteins degradation. In our study, we uncovered that HF or PEG‐HF glued integrin *β*4 and CRL4B^WDR18^ E3 ligase and the following integrin *β*4 degradation, highlighting the importance of CRL4B complex in molecular glue degraders involved targeted proteins degradation. Crucially, we identify WDR18 as a novel substrate receptor essential for this process, expanding the druggable landscape of E3 ligase components.

In summary, we found that HF exhibits high inhibitory effect on ESCC cells by acting as a molecular glue degrader to promote integrin *β*4 degradation, and inducing apoptosis. This work not only establishes a paradigm for degrading transmembrane oncoproteins via molecular glues but also unlocks WDR18 as a target for rational drug design and natural product optimization in targeted protein degradation therapeutics.

## 4 Experimental Section

### 4.1 Materials

Anti‐rabbit IgG (7074), Anti‐mouse IgG (7076), Integrin *β*4 (14803), LC3 A/B (12741), cleaved PARP (5625), HA‐tag (3724), caspase 3 (9662), cleaved caspase 3 (9661), Bcl‐2 (15071), YAP (14074), p‐YAP (13008), MST 1 (3682), LATS 1 (3477), p‐MST1 (49332), CAND1 (8759), Cul1 (4995), Cul3 (10450), Rbx1 (11922) were purchased from Cell Signaling Technology. Antibodies against DDB1 (A3827), DCAF7 (A6787), WDR18 (A15875) were purchased from ABclonal. Antibodies against *β*‐actin (60008‐1‐Ig) and GAPDH (60004‐1‐Ig), Cul4A (14851‐1‐ap), Cul4B (12916‐1‐ap) were purchased from Proteintech. Antibodies against P62 (Ab109012) and ki67 (Ab16667) were purchased from Abcam. Antibody against p‐LATS1 (YP1222) was purchased from Immunoway. Antibody against PARP (61103) was purchased from BD. Pierce^TM^ IP Lysis buffer (87788), Lipofectamine 2000 Transfection Reagent (11668019) were purchased from Thermo Fisher Scientific Inc. Halofuginone (S8144), Selleck L1400 Nature Product Library (L1400), Chloroquine (S6999), ZVAD‐fmk (S7023), Necrostatin‐1 (S8037), Ferrostatin‐1 (S7243) were purchased from Selleck Chemicals. Cycloheximide(CHX) (HY‐12320), MG132 (HY‐13259) were purchased from MCE. Halofuginone‐PEG‐biotin was modified and synthesized at Wayen Biotechnologies. SiRNAs were designed and synthesized by RIBOBIO and Tsingke.

### 4.2 Cell Lines and Cell Culture

The YES2, KYSE30, KYSE70, KYSE140, KYSE150, KYSE180, KYSE410, KYSE450, KYSE510 cells were gifts from Professor Yutaka Shimada (Kyoto University). All the ESCC cell lines were cultured in RPMI1640 (Gibco) with 10% fetal bovine serum at 37°C with 5% CO_2_. The HEK293T cell was cultured in DMEM (Gibco) with 10% fetal bovine serum at 37°C with 5% CO_2_.

### 4.3 MTS Assay

5×10^3^ ESCC cells were seeded in 96‐well culture plates, 24 hours later, different concentrations of drugs were added into cells, 72 hours later, cell viability was detected by MTS according to the manufacturer's instructions. Briefly, 10 µL MTS (15 mg/ ml) and 90 µL PBS were added to cultured cell for 2 hours in the incubated condition, 2 hours later, OD 490 value was obtained and the Inhibitory Ratio (IR) of different drugs were calculated by the following formula IR = 1‐ (OD_experiment –_ OD_blank_) / (OD _control –_ OD_blank_).

### 4.4 Colony Formation Assay

1000 drug‐exposed cells were seeded into six‐well culture plate and incubated at 37°C with 5% CO_2_ for about 10 days. And after a wash with pre‐cold PBS, cells were fixed with methanol for 20 min and stained with crystal violet for 15 min. Colonies were examined and calculated by G: box (Syngene) automatically.

### 4.5 Invasion Assay

The transwell invasion assay was performed using the transwell chamber with a matrigel‐coated filter. ESCC cells were starved in serum‐free medium for 12 h, then plated on the top chamber with or without different concentrations of drugs for 24 h, followed by removal of cells inside the upper chamber with cotton swabs, and the invasive cells on the lower side were fixed, and stained with 0.1% crystal violet solution, and counted by using microscope.

### 4.6 Cell Scratch Assay

5×10^5^ cells were seeded in a six‐well culture plate, then sterile 200‐µL pipette tip was used to generate a straight line on the plated cell in each well. PBS was used to remove the detached cells. Then serum‐free medium and different concentrations of drugs were added to cells for certain time, including 0, 2, 6, 12, 20 h time points, all of these groups were monitored and photographed at a certain time point.

### 4.7 Cell Apoptosis Assay

5×10^5^ cells were seeded in a six‐well culture plate, and then treated with different drugs, followed by propidium iodide and annexin V staining. Flow cytometry was used to determine the percentage of apoptotic cells.

### 4.8 Western Blot

Total cellular lysates were prepared in lysis buffer. Identical quantities of proteins were separated by SDS‐PAGE and then transferred onto the PVDF membranes. After the corresponding antibodies staining, the membranes were incubated with a secondary antibody. *β*‐actin and GAPDH were used as a loading control. The antibodies information is described above.

### 4.9 RNA and Truncated Plasmids Interference

For *ITGB4* knockdown and truncated *ITGB4* plasmids transfection, 1.5×10^5^ KYSE 30 or KYSE 450 cells or HEK 293T cell were seeded on six‐well plates respectively, and transfected with oligonucleotides using lipofectamine 2000 according to the manufacturer's instructions. The transfected cells were cultured at 37 °C with 5% CO_2_ for 48 h. The sequences of targeted genes and recombinant plasmids were described in Table S1.

### 4.10 DARTs Assay

Total cellular lysates were prepared in lysis buffer, 5 µL drugs with diluted concentrations were co‐cultured with 95 µL protein lysates at room temperature for 30 min, then, 20 µL protease K solution was added to drug‐protein complex to digest the uncoupled proteins at room temperature for 20 min, followed by addition of protease inhibitor to stop protease K activity. Finally, integrin *β*4 abundance was detected in the drug‐ protein‐protease K complex by western blot.

### 4.11 SPR Assay

Integrin *α*6*β*4 protein was diluted to 1mg/ mL with SPR buffer (0.1 M HEPES, 0.15 M NaCl, 0.05% tween 20, 2 mM MgCl_2_, 2 mM MnCl_2_ and 5% DMSO), and attached to GE‐CM5 microarray. Then, series diluted HF with concentration ranging from 0 to 200 µM were controlled to flow through the surface of the protein‐binding micro array, the binding affinity was monitored and analyzed by Biacore S200.

### 4.12 PDX Model Creation

Collect and cut the fresh ESCC tumor tissue during surgery into 3–5 mm^3^ volumes and implant them subcutaneously in the lateral abdomen of NGS mice. When the volume reaches about 150 mm^3^, mice were randomly divided into two groups based on the tumor size to ensure no difference in tumor size between the groups, among which, one group mice were treated with HF by gavage at the dosage of 0.5 mg / kg/ 100 µL, while the other group mice were treated with 0.9% Nacl instead. Tumor size was measured using caliper, and calculated by using the formular (L×W^2^)/2. The clinical operation was approved by the medical ethics committee of Peking University Cancer Hospital & institute. All animal experiments were approved by the Institutional Animal Care and Use Committee of Peking University Cancer Hospital & institute.

### 4.13 Tumor Xenograft Model

5×10^6^ KYSE 450 cell was subcutaneously injected into the right axilla of 6‐week‐old female nude mice. When the volume reaches about 150 mm^3^, mice were randomly divided into eight groups based on the tumor size to ensure no difference in tumor size between the groups, including group treated with 0.9% Nacl, group with biotin&PEG exposure, HF exposed groups with low dosage administration (0.25 mg/ kg), medium dosage administration (0.5 mg/ kg), and high dosage administration (1 mg/ kg), as well as PEG‐HF exposed groups with low dosage administration (0.25 mg/ kg), medium dosage administration (0.5 mg/ kg), and high dosage administration (1 mg/ kg). Tumor size was measured using caliper, and calculated by using the formular (L×W^2^)/2. Before mice sacrifice, mice blood in each group were collected from the eyeballs, and serum biochemical index of those tumor bearing mice, including Total protein, Albumin, Creatinine, Urea, Aspertate Aminotransferase, Alanine Transaminase, Direct Bilirubin and γ‐Glutamyltransferase were checked in the commercial market.

### 4.14 Immunohistochemical (IHC) Stanning

Tissues were fixed, deparaffinized, and hydrated, and their antigens were retrieved using EDTA antigenic retrieval buffer by boiling the tissues in a pressure cooker for 10 min. Then, the slides were blocked using 10% goat serum for 40 min at room temperature, labeled overnight at 4°C with an anti‐integrin *β*4 antibody and an anti‐Ki‐67 antibody, tagged with a universal secondary antibody for 40 min at room temperature, and stained with 1× DAB solution. A tissue microarray containing 107 dots of ESCC tissue and 72 dots of adjacent tissues was purchased from Shanghai Outdo Biotech Co., Ltd. (Shanghai, China). To conduct IHC analysis of the tissue microarray, tissue specimens were subjected to immunostaining using an anti‐integrin *β*4 antibody. The Institutional Research Ethics Committee provided ethical approval and documented informed consent for all patients.

### 4.15 Statistical Analysis

IBM SPSS version 25 software packages was used to assess the differences between experimental groups. Data in vitro experiments were analyzed by two‐tailed Student's t test. In vivo experiments, differences in tumor growth between groups were statistically analyzed using analysis of variance. The relationship between clinical pathological features was statistically analyzed using Chi‐square test. All experimental data presented in this study are means ± SEM. In all experiments, differences were considered to be significant when P was less than 0.05. *P < 0.05, **P < 0.01, ***P < 0.001. All vitro assays were repeated at least three times.

## Author Contributions

Q.Z., W.Z., W.G., and D.Y. performed conceptualization. W.G. and D.Y. performed methodology. Q.Z. and W.Z. performed investigation. J.T., Y.C., and T.Y. performed visualization. Q.Z and W.Z. performed supervision. W.G. and D.Y. wrote the final manuscript. W.Z. and Q.Z. Wrote, review and edited the final manuscript.

## Conflicts of Interest

The authors declare no conflict of interest.

## Supporting information




**Supporting File**: advs74835‐sup‐0001‐SuppMat.docx.

## Data Availability

The data that support the findings of this study are available from the corresponding author upon reasonable request.
