# Supplementary material for: Halofuginone is a Molecular Glue Degrader of Integrin β4
Source: Adv Sci (Weinh). 2026 Mar 24;13(30):e15970. doi: 10.1002/advs.202515970 (PMC13248763; doi:10.1002/advs.202515970)
Supplement: Supplementary file 1 — Supporting File: advs74835‐sup‐0001‐SuppMat.docx. [file ADVS-13-e15970-s001.docx]

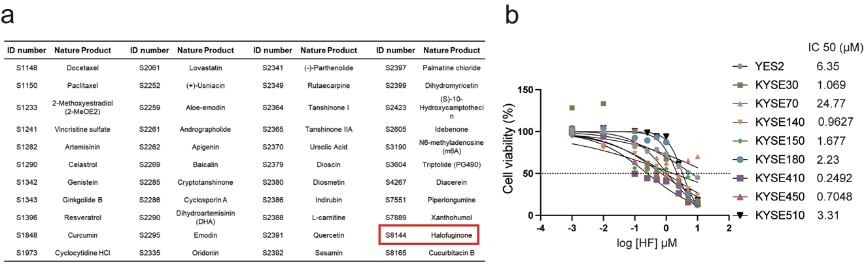


Fig. S1. **Candidate molecules screening from a natural product library.**

a. At a concentration of 10 μM, 44 molecules from the natural product library that exhibit an inhibitory rate exceeding 50%. b. The IC50 value of HF to different ESCC cell lines.


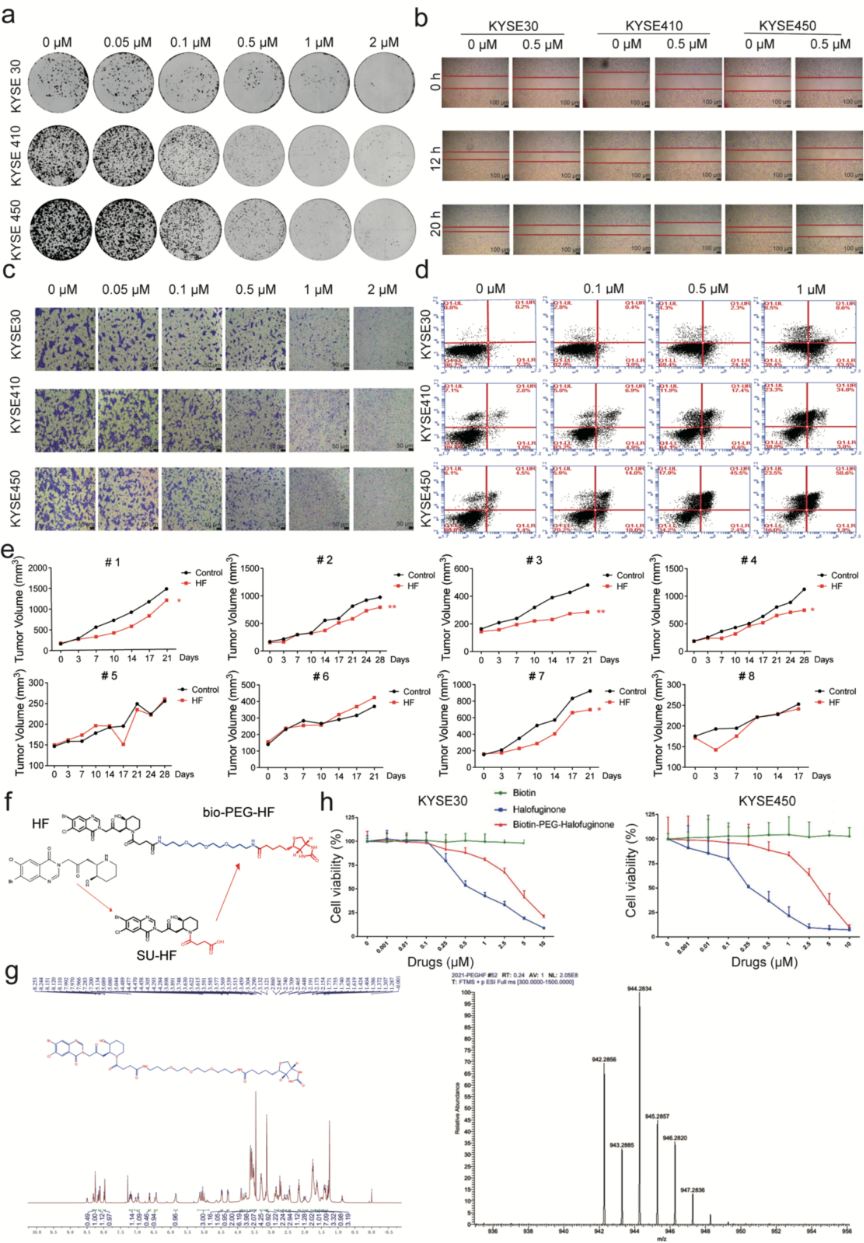


Fig. S2. **HF exhibits cytotoxicity to ESCC cells.**

1. d. HF administration inhibits ESCC aggressive phenotypes (Colony formation in Fig. S2a, Migration ability in Fig. S2b, Invasion ability in Fig. S2c, Apoptosis in Fig. S2d) on a dose-dependent manner in KYSE30, KYSE410 and KYSE450 cells. e. Tumor volumes of the eight PDX model. f. Schematic diagram of HF PEGylation, and structures of HF, SU-HF (midbody) and PEG-HF with a biotin. g. H NRM and HPLC-Mass spectrometry identification of PEG-HF. h. MTS assay to determine the difference of HF, PEG-HF exposure to cell viability in KYSE30 and KYSE450 cells.


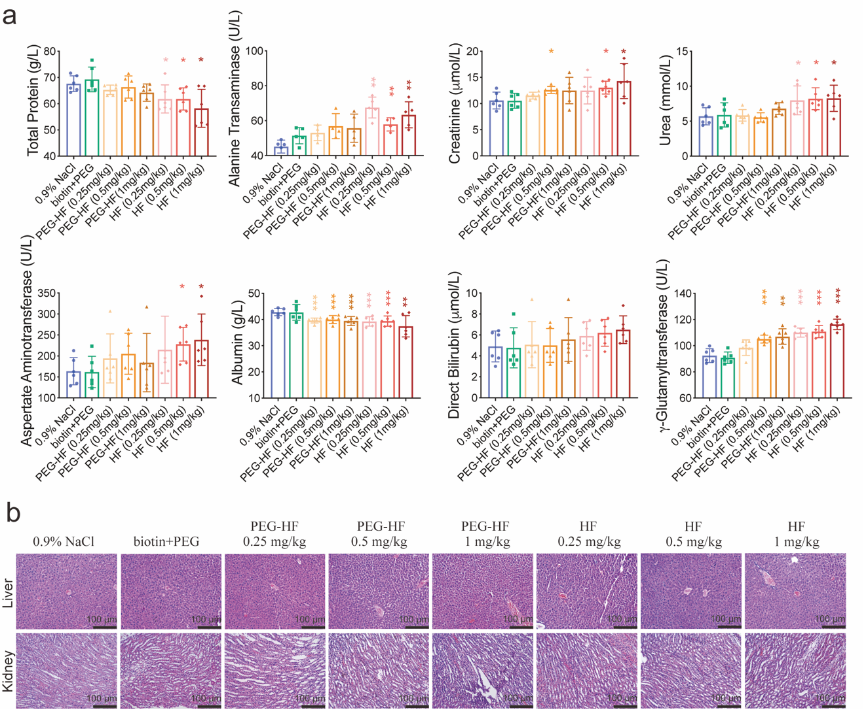


Fig. S3. **PEG-HF treatment exhibits low side effect compared to HF administration.**

a. Serum biochemical index analysis of those tumor bearing mice, including total protein, albumin, creatinine, urea, aspertate aminotransferase, alanine transaminase, direct Bilirubin and γ-Glutamyltransferase, in these groups (n=6). b. HE staining of liver and kidney tissue in different groups, the scale bar is 100 μm (n=6). All data are presented as mean ± s.d. Statistical significance was determined by a two-tailed Student’s t-test. *P < 0.05, **P < 0.01, ***P < 0.001.


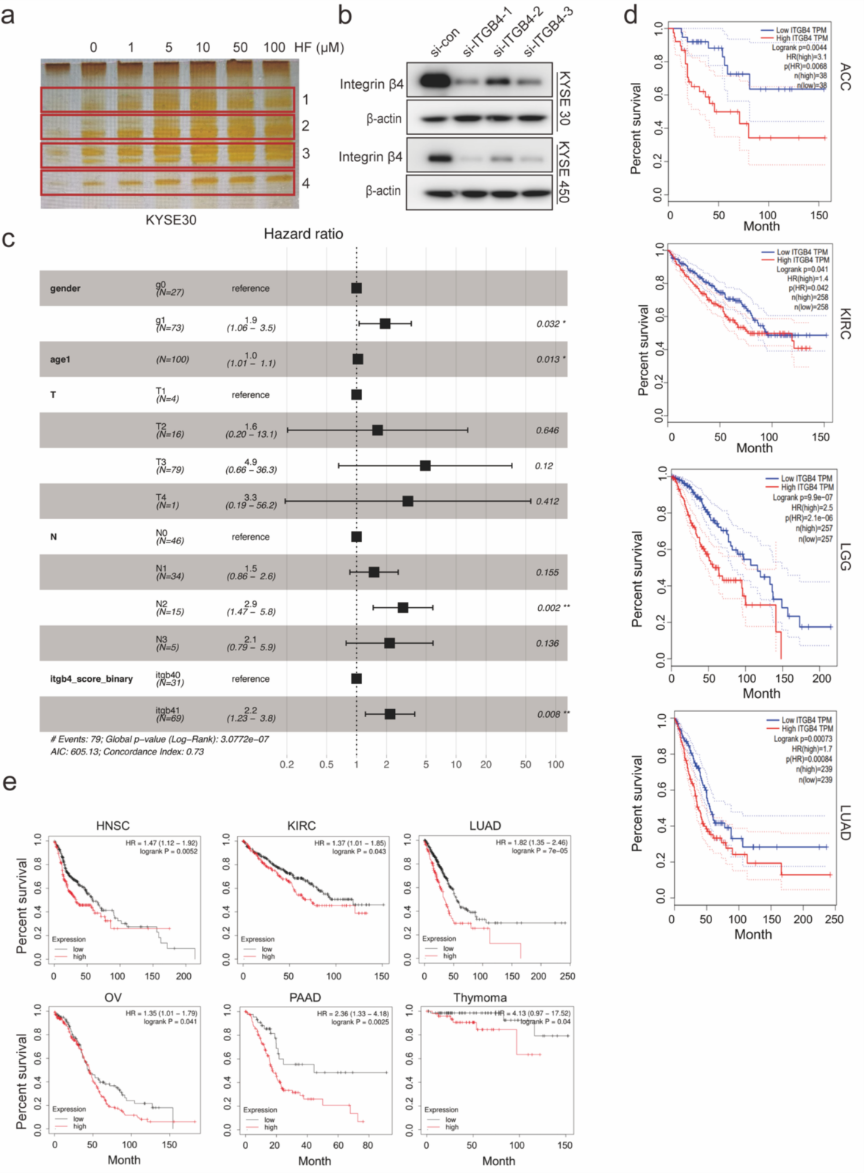


Fig. S4. **High *ITGB4* expression correlates with poor prognosis in various cancer types.**

Silver staining of samples oriented from DARTs assay. b. Western blot analysis confirmed the knockdown efficiency of *ITGB4* targeting siRNA. c. Cox regression analysis to test whether integrin β4 can be served as an independent influencing factor for survival prognosis. d-e. Database oriented data analyses of the correlation of high expression of *ITGB4* with survival rates in solid tumors including ACC, KIRC, LGG, LUAD (data collecting in GEPIA database), Glioma, HNSCC, Ovarian cancer, Pancreatic cancer, Lung cancer, Renal cancer (data collecting in KM-plotter database).


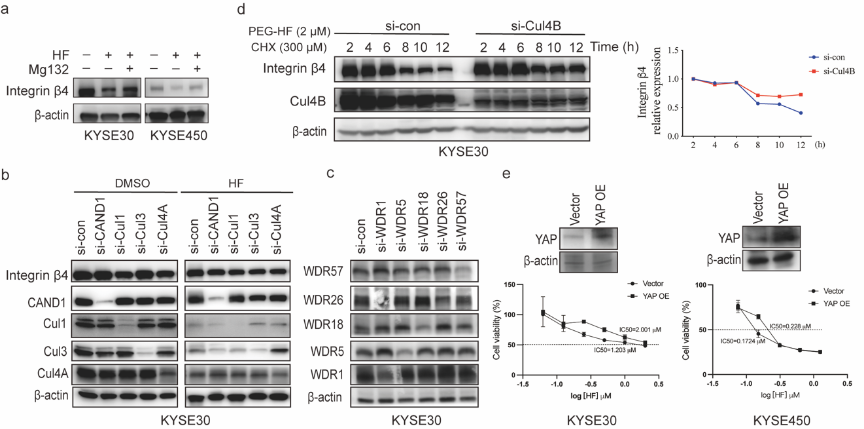


Fig. S5. **CRL4B^WDR18^ E3 ligase matters in mediating integrin β4 degradation upon HF exposure.**

a. The proteasome inhibitor Mg132 was added to ESCC cells to test whether proteasome pathway inhibition prevented integrin β4 from degradation upon HF exposure. b. Western blot analysis of integrin β4 abundance after knockdown of cullin associated proteins. c. Western blot analysis of knockdown efficiency of WDR proteins. d. Western blot was performed to determine the degradation rate of integrin β4 after knockdown of Cul4B in PEG-HF administrated KYSE30 cell. e. HF was added to YAP-overexpressing ESCC cells, then MTS assay was performed to test the cell viability and the following IC50 value calculation of HF in these cells.

**Table S1** siRNA sequences and recombinant plasmids used in this study

| siRNA sequences | target sequence (5’ to 3’) |
| --- | --- |
| si-ITGB4-1 | ACGACAGCTTCCTTATGTA |
| si-ITGB4-2 | CAGCGACTACACTATTGGA |
| si-ITGB4-3 | CACACGGGACTACAACTCA |
| si-CAND1-1 | GAAGCAAACTCGTCCTGTA |
| si-CAND1-2 | GGTTCTGACTTGCCTAATA |
| si-DDB1 | GTCTGTTGAGCGACCGTAA |
| si-CUL1 | CAGCATGATCTCCAAGTTA |
| si-CUL3 | GCACATGAAGACTATAGTA |
| si-CUL4A | CGTTTAGAGAAGACTCACT |
| si-CUL4B | GATAGAAGTTTACTTCGAA |
| si-DCAF7 | GATACGACATGCACCATCT |
| si-Rbx1 | GGGATATTGTGGTTGATAA |
| si-WDR1 | GATTTACGCAATTAGTTGG |
| si-WDR5 | CGCTCATCGATGACGACAA |
| si-WDR18 | CACCCAATGGTCTCTACGT |
| si-WDR26 | CCGATCTAAACTATTGGAT |
| si-WDR57 | GGACATACTTCCTTTGTGA |
| Recombinant DNA | Sequence from 5’ to 3’ |
| ITGB4-C1 | from 734 aa to 1100 aa |
| ITGB4-C2 | from 1101 aa to 1400 aa |
| ITGB4-C3 | from 1401 aa to 1822 aa |

| Clinicopathological Features | | Total cases | Integrin β4 expression | | χ2 | p Value |
| --- | --- | --- | --- | --- | --- | --- |
|  |  |  | Low expression (0-6） | High expression  (7-12) |  |  |
| Gender | Male | 82 | 23 | 59 | 1.861 | 0.173 |
|  | Female | 26 | 11 | 15 |  |  |
| Age | ＜65 | 46 | 11 | 35 | 1.816 | 0.178 |
|  | ≥65 | 61 | 22 | 39 |  |  |
| Survival | ＜5 years | 83 | 19 | 64 | 13.472 | **0.000***** |
|  | ≥5 years | 24 | 15 | 9 |  |  |
| T_Stage | T1+T2 | 20 | 7 | 13 | 0.152 | 0.697 |
|  | T3+T4 | 82 | 25 | 57 |  |  |
| N_Stage | N0+N1 | 86 | 29 | 57 | 1.704 | 0.192 |
|  | N2+N3 | 21 | 4 | 17 |  |  |
| LNM | No | 49 | 20 | 29 | 4.217 | **0.040*** |
|  | Yes | 58 | 13 | 45 |  |  |
| PD-L1 | Low (＜2) | 42 | 14 | 28 | 0.09 | 0.746 |
|  | High (≥2) | 59 | 18 | 41 |  |  |
| CD8+ | Negtive (＜5%） | 45 | 12 | 33 | 1.207 | 0.272 |
|  | Positive （≥5%） | 54 | 20 | 34 |  |  |

**Table S2** The relationship between Integrin β4 expression and clinicopathologic features
